# Periodontitis and cardiovascular diseases: bridging the gap through mitochondrial dysfunction

**DOI:** 10.3389/froh.2026.1791758

**Published:** 2026-04-28

**Authors:** Weiquan Li, Mingshu Huang, Dan Lu, Ping Li, An Li, Shulan Xu

**Affiliations:** 1 Center of Oral Implantology, Stomatological Hospital, School of Stomatology, Southern Medical University, Guangzhou, China; 2 Department of Neurology and Stroke Center, The First Affiliated Hospital of Jinan University, Guangzhou, China; 3 Clinical Neuroscience Institute, The First Affiliated Hospital of Jinan University, Guangzhou, China; 4 School and Hospital of Stomatology, Guangdong Engineering Research Center of Oral Restoration and Reconstruction, Guangzhou Medical University, Guangzhou, China; 5 Guangzhou Key Laboratory of Basic and Applied Research of Oral Regenerative Medicine, Guangzhou Medical University, Guangzhou, China; 6 Department of Periodontology, Stomatological Hospital, School of Stomatology, Southern Medical University, Guangzhou, China; 7 Center for Dentistry and Oral Hygiene, University Medical Center Groningen, University of Groningen, Groningen, Netherlands

**Keywords:** cardiovascular diseases, mitochondrial dysfunction, oxidative stress, periodontitis, systemic inflammation

## Abstract

Epidemiological studies have reported a distinct link between periodontitis and cardiovascular diseases (CVD). However, the underlying mechanisms—except for systemic inflammation—are still poorly understood. This review examines the evidence that mitochondrial dysfunction represents a key pathological denominator in both periodontitis and CVD. We explain the mechanisms through which dysbiotic periodontal microenvironment and related inflammation can disrupt key mitochondrial functions, including redox balance and quality control. This disruption can exacerbate local tissue damage in the oral cavity and contribute to CVD. Important mechanisms include abnormal mitochondrial dynamics, impaired mitophagy, and impaired mitochondrial biogenesis, which are involved in both periodontitis and CVD. Examining the relationship through the perspective of mitochondrial dysfunction may elucidate the underlying mechanism and allow for new treatment possibilities. Targeting these shared mitochondrial pathways may unveil new strategies to reduce the impact of both periodontitis and CVD.

## 1 Introduction

**Figure 1:**
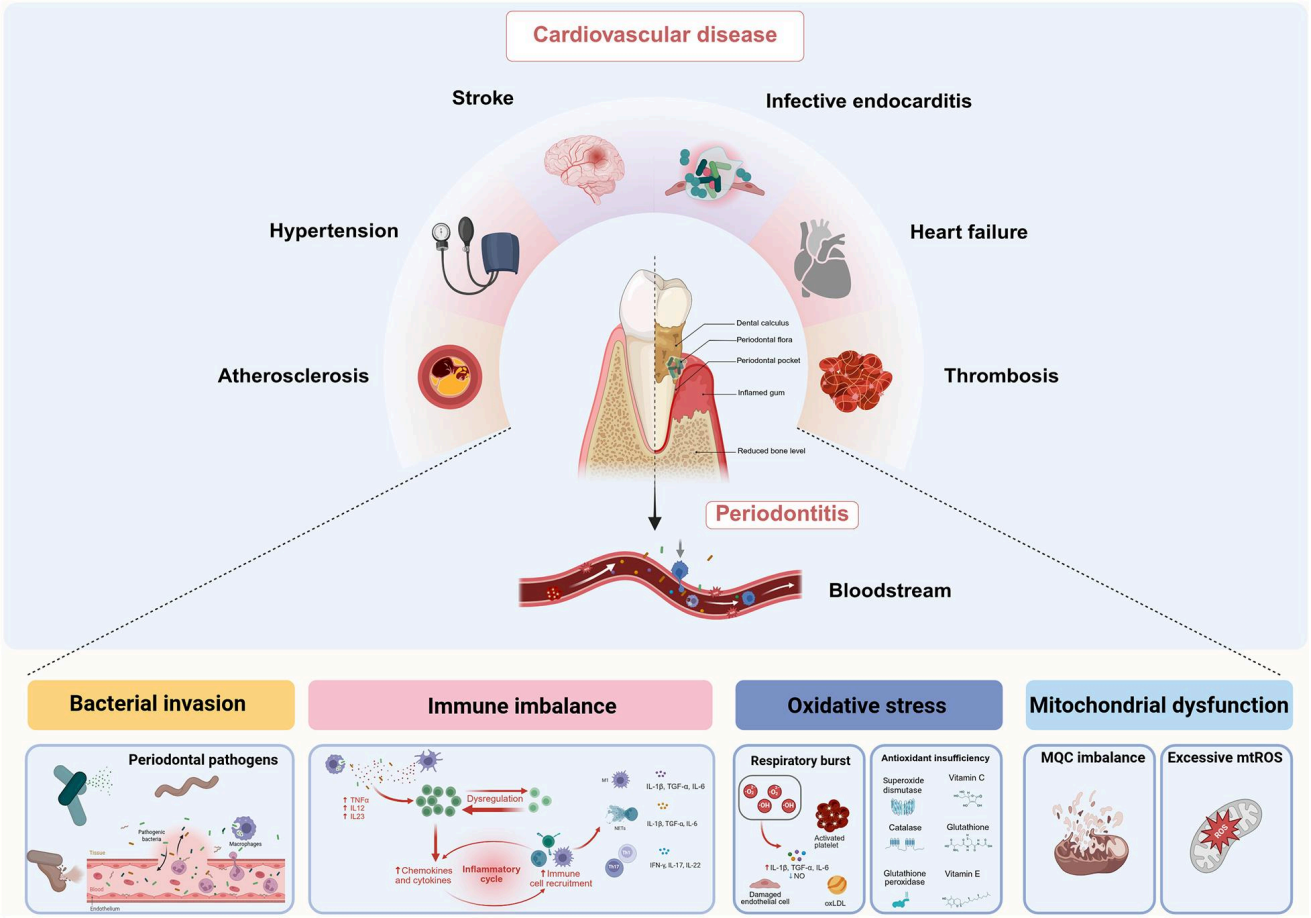
Mechanisms that establish a link between periodontitis and cardiovascular disease. Periodontopathogens enter the bloodstream and contribute to cardiovascular disease through several key pathways, including bacterial dissemination, immune imbalance, oxidative stress, and mitochondrial dysfunction (16, 17). (Created in BioRender).

Periodontitis and cardiovascular disease (CVD) are two common chronic conditions that pose a significant threat to global health. Periodontitis is an inflammatory disease caused by bacterial biofilms, leading to the destruction of tooth-supporting structures. It affects more than 1 billion individuals globally in 2021 (1, 2). CVD encompasses a range of disorders of the heart and blood vessels, mainly comprising coronary heart disease (CHD), cerebrovascular disease, and peripheral arterial disease (3). As a leading cause of death worldwide, CVD accounts for approximately 18 million deaths annually (4). Extensive cohort studies have confirmed a significant association between periodontitis and CVD. Patients with periodontitis exhibit a markedly increased risk of developing conditions such as atherosclerosis (5), peripheral arterial disease (5), myocardial infarction (MI) (6), CHD (7), carotid calcifications (8), and stroke (9). In addition, periodontal pathogens have been directly detected in cardiovascular lesions (10); genomic analyses have revealed shared susceptibility gene variants between the two conditions (11, 12). More importantly, periodontal therapy has been proven effective in mitigating the advancement of CVD (13). A randomized controlled trial (RCT) demonstrated that intensive periodontal treatment (IPT) significantly reduced ambulatory 24-h blood pressure in hypertensive patients, with a remarkable average decrease of 11.1 mmHg in systolic blood pressure (14). Building on this, another RCT found that IPT notably slowed the progression of carotid intima-media thickness in patients with periodontitis who had no other underlying conditions (15). This indicates that periodontitis is not only a contributing factor to hypertension but also that its effective treatment can provide long-term cardiovascular benefits, ranging from improved vascular function to delayed arterial hardening (Figure 1).

It is known that periodontitis and CVD share a range of risk factors, including age, smoking, obesity, and diabetes (18, 19). The pathogenesis and progression of both conditions are often explained by the systemic inflammation driven by these factors. Even after adjusting for traditional shared risk factors, periodontitis remains significantly associated with the incidence of CVD (19). This suggests a more direct link between periodontitis and CVD. It posits that periodontal pathogens transfer into the bloodstream systematically amplify the inflammatory response by stimulating circulating inflammatory mediators, thereby increasing the risk of cardiovascular events (16). A fundamental question persists: how does a localized oral bacteria instigate and exacerbate pathological changes in the distant cardiovascular system?

In the classic hypothesis, periodontal pathogens and recurrent bacteremia activate monocytes/macrophages and vascular endothelial cells (ECs), leading to sustained increases in inflammatory mediators such as C-reactive protein, TNF-α, il-1β, and IL-6 (16). This bacteremia-systemic inflammation model offers a partial explanation for the epidemiological association between periodontitis and CVD. However, a framework focused on circulating inflammatory mediators still does not resolve several key issues. It does not clarify why chronic oral inflammation in some individuals results in sustained, organ-specific injury within the cardiovascular system. These gaps suggest that an inflammation-focused view is not sufficient. More fundamental cellular mechanisms shared across chronic diseases should be taken into account.

In this context, mitochondrial dysfunction has emerged as a key pathological node that may link periodontitis and CVD (20, 21). As a central organelle regulating redox balance, energy metabolism, and mitochondrial quality control (MQC), mitochondria show similar alterations across chronic inflammatory conditions, including excessive reactive oxygen species (ROS) production, disturbed mitochondrial dynamics, impaired mitophagy, and reduced mitochondrial biogenesis (mitobiogenesis). Recent evidence supports an integrative hypothesis in which periodontal pathogens and their virulence factors disrupt these mitochondrial processes, promoting persistent local inflammation and tissue destruction in the gingiva while also propagating aberrant mitochondrial signals systemically, thereby contributing to cardiovascular pathology (17, 22).

Building on this concept, the present review examines the putative link between periodontitis and CVD from the perspective of mitochondrial dysfunction. We first provide a brief overview of mitochondrial structure and core functions, then summarize mitochondrial abnormalities in periodontitis and in CVD, and discuss mitochondrial crosstalk with other organelles and its role in immunometabolic reprogramming. Finally, we highlight shared and disease-specific patterns of mitochondrial dysfunction in these two conditions and explore potential mitochondria-targeted interventions and their implications for integrated periodontal-cardiovascular management.

## 2 Basic structure and function of mitochondria

Mitochondria are key metabolic organelles distributed throughout the cytoplasm as spherical or short rod-shaped structures under physiological conditions (23). Mitochondria can dynamically change their morphology and function in response to the cell's energy needs, a feature known as mitochondrial heterogeneity, through fission, fusion, cristae remodeling, changes in matrix content, and interactions with other organelles like the endoplasmic reticulum (ER) (24). Mitochondria are double-membraned organelles, and their inner membrane folds inward to form cristae, significantly increasing the surface area (25). Proteins and molecules embedded in these membranes have a critical role in mitochondrial functions (26). Abnormalities in mitochondrial morphology and structure, including swelling, disrupted cristae, matrix vacuolation, and electron-dense deposits, are associated with various human diseases (25).

Mitochondria play a critical role in energy production, and their dysfunction severely disrupts cellular metabolism. In eukaryotic cells, mitochondria generate energy through the electron transport chain (ETC), which includes four protein complexes (I to IV), electron carriers, and cytochromes (27). Complex I (NADH dehydrogenase) and Complex II (succinate dehydrogenase) are mitochondrial membrane protein complexes that catalyze the transfer of electrons from different substrates to ubiquinone (28, 29). Meanwhile, Complex III (cytochrome c reductase) and Complex IV (cytochrome c oxidase) accept these electrons from ubiquinone and relay them via cytochrome c, ultimately to molecular oxygen, which is reduced to water (27). During electron transfer, adenosine diphosphate (ADP) is converted into adenosine triphosphate (ATP) through oxidative phosphorylation (OXPHOS) (27). Electrons and protons can leak from the ETC under normal conditions, resulting in incomplete electron transfer. These electrons interact with oxygen, generating superoxide, which is further converted into hydrogen peroxide and other ROS—collectively called mitochondrial reactive oxygen species (mtROS) (30). The principal site of mtROS generation is complex I, which produces ROS at functional sites such as I_F_ and I_Q_ (31). Although complexes II and III also produce small amounts of ROS, their contribution is much smaller. Consequently, mitochondria exist in a precarious balance: they are both the source of cellular energy and a potential trigger of oxidative stress, as well as an important signaling hub.

**Figure 2:**
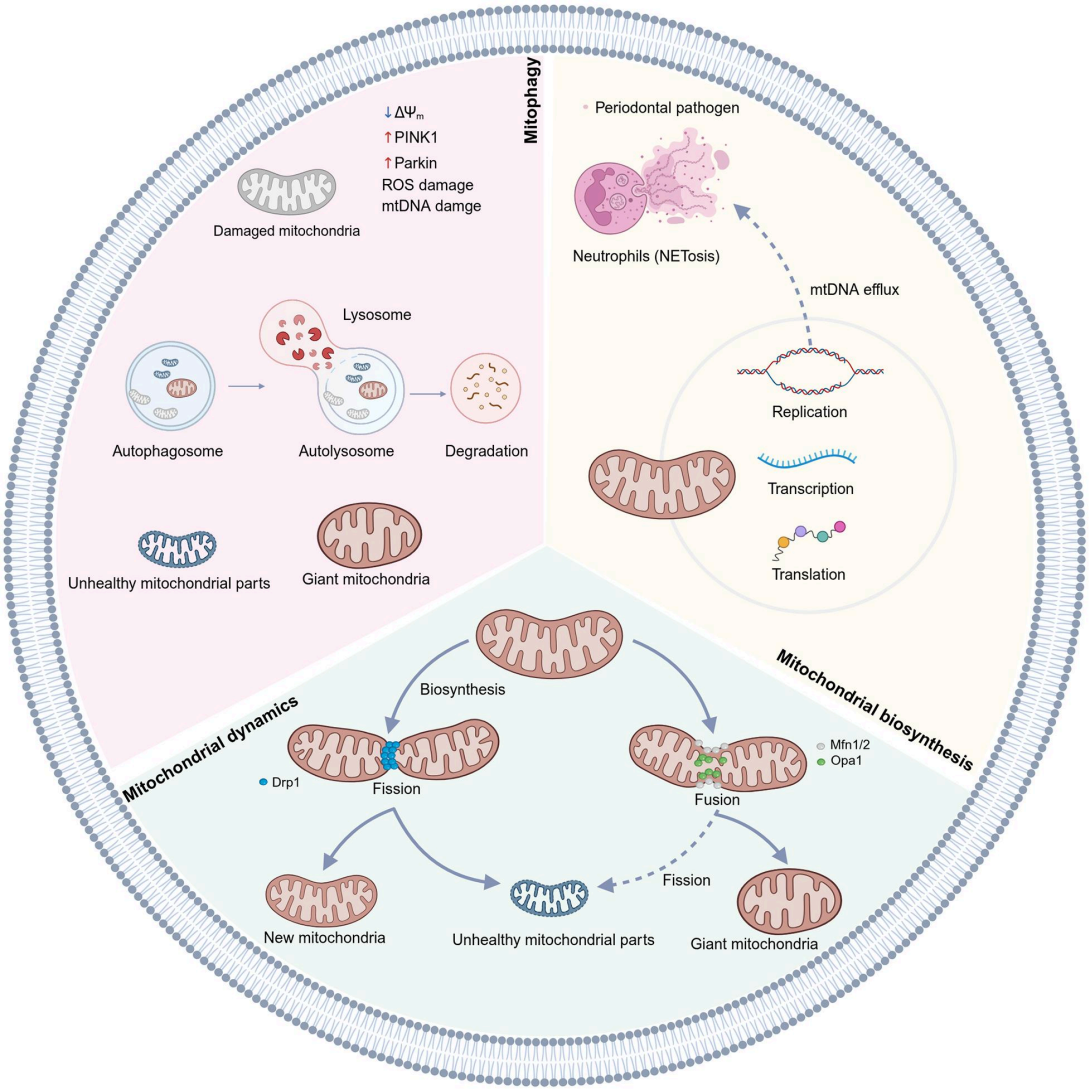
Key mechanisms of mitochondrial quality control. Mitochondrial quality is maintained by biogenesis (new synthesis), dynamics (fission and fusion), and mitophagy (elimination of damaged mitochondria) (32). Fusion is mediated by proteins such as Mfn1/2 and OPA1, while fission is controlled by Drp1. The PINK1-Parkin pathway marks damaged mitochondria for lysosomal degradation via mitophagy. Mitochondria have their own DNA, and under stress, mtDNA can be released to trigger immune responses. (Created in BioRender).

To maintain this balance, cells have evolved a sophisticated MQC system. This system encompasses dynamics [fission mediated by dynamin-related protein 1 (Drp1) and fusion mediated by mitofusin (Mfn)1/2 and Optic Atrophy Protein 1 (OPA1)], mitophagy [including pathways such as PTEN-induced putative kinase 1 (PINK1)- Parkin protein (Parkin) and BCL2/adenovirus E1B 19 kDa protein-interacting protein 3-like (BNIP3L)/NIX], and biogenesis [which involves mitochondrial DNA (mtDNA)-encoded and nuclear-encoded protein synthesis] (32). MQC are crucial for maintaining cellular balance, regulating cell death, handling calcium, and mediating intracellular signaling (Figure 2) (33).

## 3 Role of mitochondria in periodontitis

**Figure 3:**
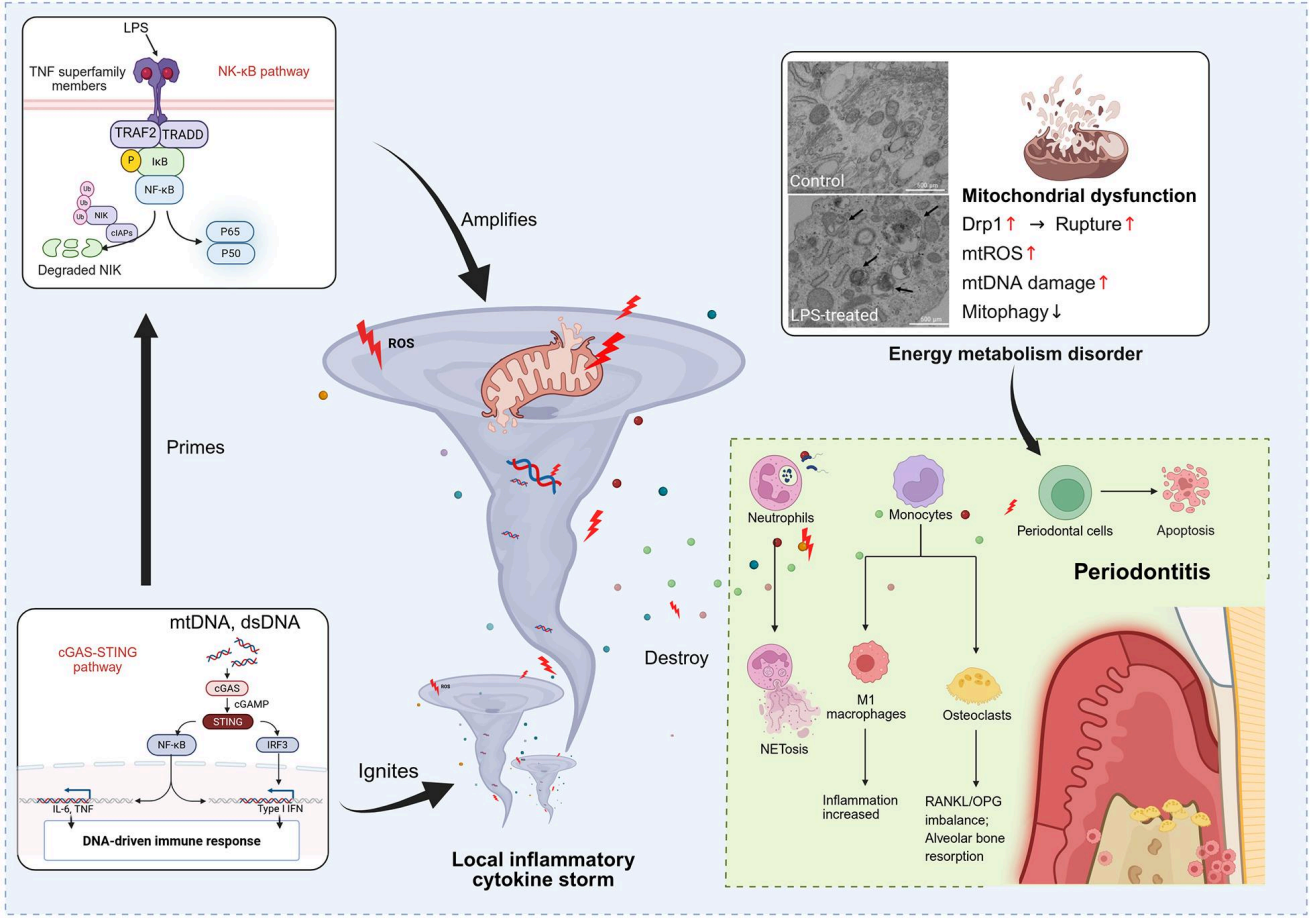
Mitochondrial dysfunction promotes periodontal inflammation. LPS stimulation in periodontal tissue induces mitochondrial damage in immune cells, leading to excessive mtROS production, mtDNA release, and abnormal fission (21). These mitochondrial components activate NF-κB and cGAS-STING pathways, increasing production of proinflammatory factors (e.g., IL-6, TNF-α) and sustaining a local inflammatory cycle (34). This persistent inflammation disrupts bone metabolism, promotes M1 macrophage polarization, and enhances NET formation, collectively accelerating periodontal tissue destruction (35, 36). (Created in BioRender). The mitochondrial electron microscope image in this schematic diagram has been reproduced from “Ultrastructure of lipopolysaccharide-treated human gingival fibroblasts (10 μg/mL). Control fibroblasts show mitochondria with typical ultrastructure. Laminar bodies and autophagosome with mitochondria were present in LPS-treated fibroblasts (black arrow); Bar = 500 nm. LPS: lipopolysaccharide” by Pedro Bullon, Mario David Cordero, José Luis Quiles, Maria del Carmen Ramirez-Tortosa, Adrian Gonzalez-Alonso, Simona Alfonsi, Rocio García-Marín, Manuel de Miguel and Maurizio Battino, licensed under CC BY.

Mitochondria, which are crucial for energy metabolism and maintaining redox balance, play a significant role in the development of periodontitis. Dysbiotic periodontal microenvironment can cause mitochondrial dysfunction, resulting in increased ROS production, altered mitochondrial dynamics, and defective mitophagy, exacerbating local inflammation and tissue damage. This section examines the processes (including cristae dynamics, oxidative stress, mitophagy, and biogenesis) by which mitochondria contribute to the progression of periodontitis (Figure 3).

### 3.1 Oxidative stress in periodontitis

As the primary site of OXPHOS, mitochondrial cristae provide insights into the pathogenesis of periodontitis. Cristae adjust their shape and number in response to cellular energy requirements and to facilitate specific biochemical processes (38, 39). Cristae often exhibit abnormalities, including loss, swelling, vacuolation, paracrystalline inclusions, simplified membranes, or onion-like structures (40, 41).

The ETC, located on the cristae membrane, plays a dual role as the common source of both cellular energy and oxidative stress. In periodontitis, bacterial Lipopolysaccharide (LPS) activates the Toll-like receptors (TLR)4/NADPH oxidase axis, triggering a burst of ROS and activating the nuclear factor kappa-B (NF-κB) pathway, ultimately driving inflammation and bone loss (42). ROS also compromise the epithelial barrier via JNK signaling and reduce E-cadherin, facilitating bacterial dissemination (43).Shared NF-κB activation may help explain the relationship between periodontitis and CVD. Despite NF-κB involvement in both periodontitis and CVD, direct evidence demonstrating that periodontitis specifically employs this pathway to induce remote vascular damage is still absent. Consequently, it remains unsubstantiated to directly label it a shared pathway.

**Figure 4:**
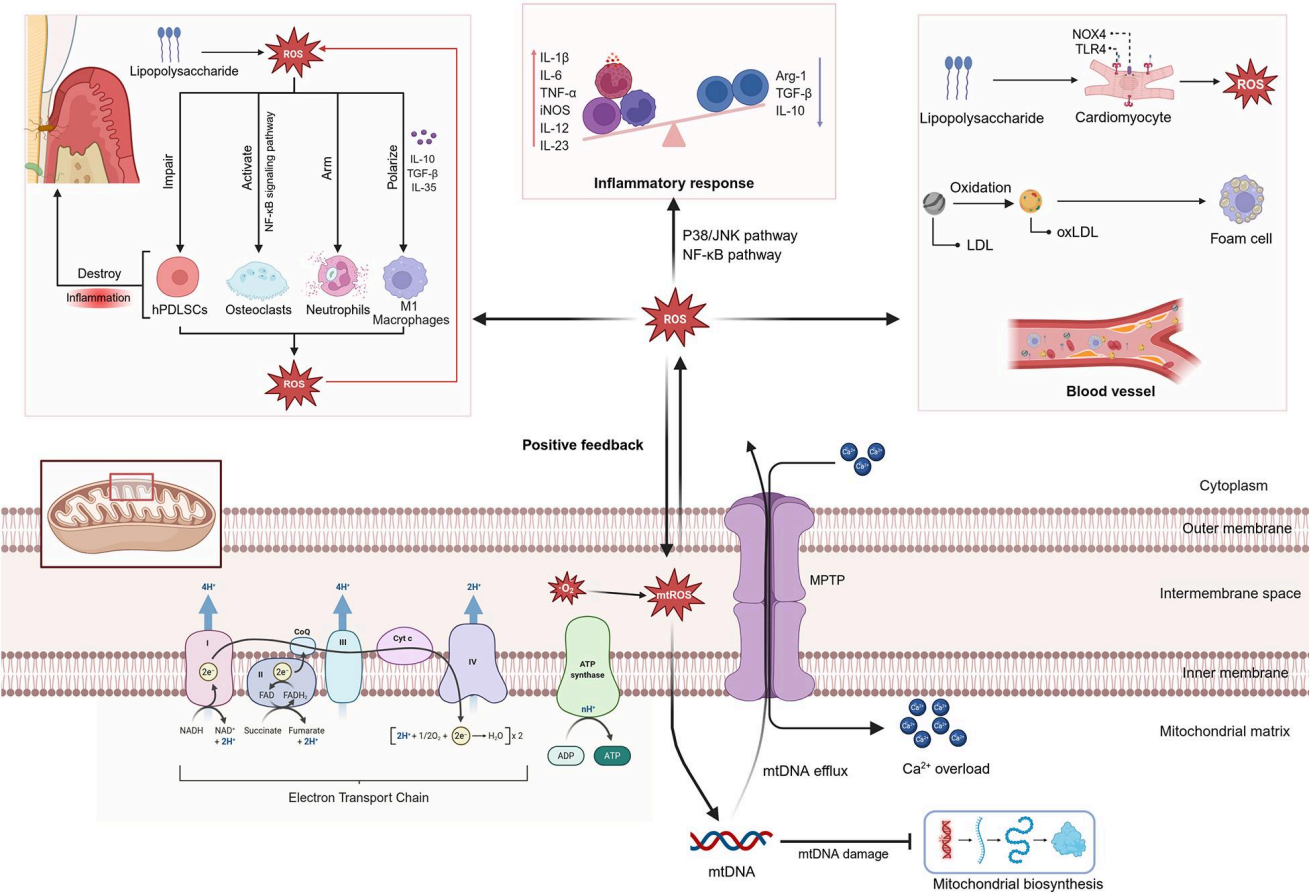
Mechanisms of mtROS-induced damage in periodontitis and CVD. mtROS produced by mitochondrial electron transport promote mtDNA damage and mPTP opening, causing calcium overload and dysfunction (50). Released mtDNA activates inflammatory pathways, increasing cytokines such as IL-1β, IL-6, and TNF-α, which further amplify mtROS generation (51). In periodontitis (left), pathogens increase oxidative stress, impair hPDLCs function, and activate NF-κB, thereby stimulating bone loss (46). In CVD (right), factors like TLR4 activation induce ROS, oxidize LDL, and promote foam cell formation in early atherosclerosis (52). (Created in BioRender).

Mitochondrial oxidative stress in host cells is a key part connecting pathogen attack to tissue destruction during periodontitis. In periodontal connective tissue, the mitochondria of human gingival fibroblasts (hGFs) and periodontal ligament cells (PDLCs) are key targets of the inflammatory response. Following *P. gingivalis*-LPS stimulation, hGFs mount a substantial mtROS burst that precedes inflammatory factor expression (44). This mtROS exacerbates oxidative stress, leading to reduced mitochondrial membrane potential and morphological abnormalities in hGFs (45). PDLCs in an inflammatory environment also display increased mtROS, decreased ATP production, and mitochondrial fragmentation (46). This mitochondrial damage directly impairs tissue repair capacity. In periodontal ligament stem cells (PDLSCs) responsible for regeneration, local oxidative stress induces excessive mitochondrial fission and apoptosis (47, 48), while concurrently suppressing peroxisome proliferator activator receptor gamma-coactivator 1α (PGC-1α) synthesis, ultimately leading to impaired osteogenic differentiation capacity; While in PDLCs, it compromises osteogenic differentiation (49). Antioxidant intervention not only rescues mitochondrial function and increases ATP production, but also restores the osteogenic capacity of PDLCs (49). These viewpoints collectively affirme the central role of mitochondria-mediated oxidative stress in periodontal pathology. Furthermore, the current understanding of cristae abnormalities and oxidative stress in periodontitis remains largely descriptive. It lacks mechanistic insights into how these ultrastructural changes directly contribute to the transition from local inflammation to systemic tissue damage (Figure 4).

### 3.2 Mitochondrial dynamics in periodontitis

Mitochondrial dynamics is an adaptive response to alterations in the cellular environment, serving to maintain cellular homeostasis by regulating mitochondrial quantity and quality (53). Under pathological conditions, dysregulated mitochondrial fission contributes not only to organelle dysfunction but also to the amplification of inflammation. Multiple evidence establishes the central role of Drp1 in this process: increased Drp1 expression has been observed in both PDLSCs and hPDLCs following hydrogen peroxide treatment (46, 54). The upregulation of Drp1 is accompanied by reduced Mfn1/2 levels and ROS crosstalk, ultimately leading to cellular apoptosis, suggesting that aberrant mitochondrial fission may critically influence cell survival (55). Notably, application of antioxidants has been shown to restore normal mitochondrial physiological activity and reduce inflammation levels in experimental cells (54).

Beyond these effects, disturbances in mitochondrial dynamics may interfere with other cellular functions, including cytokine secretion, calcium release, and macrophage activation (56, 57). Among these, Mfn2 has garnered the most attention for its role in regulating calcium signaling within the mitochondria-ER structure (58). According to previous studies, *P. gingivalis*-LPS upregulates Drp1 and phospho-Drp1 (Ser616) while downregulating Mfn2 in murine gingival tissue and hGFs, shortening or fragmenting mitochondria (59). The above findings establish Drp1-driven mitochondrial fission as a key mechanism in periodontitis-associated inflammation. Current research remains confined to cellular and animal models. The definitive role of Drp1 in driving periodontal bone resorption within the complex human physiological environment still requires verification. Moreover, the specific mechanistic relationship between Drp1 and inflammation remains unclear, and whether targeted inhibition of Drp1 could effectively constrain inflammatory responses awaits further investigation.

### 3.3 Mitophagy impairment in periodontitis pathogenesis

Damaged mitochondria are normally eliminated through programmed mitophagy to prevent the excessive release of mtROS (60). Defective mitophagy may represent a mechanism contributing to pathological tissue destruction in periodontitis. Human histology studies demonstrate a reduction in the levels of mitophagy-related proteins (e.g., PINK1 and Parkin) in the gingival tissues of periodontitis patients compared to healthy subjects (61). This observational association in human tissues is corroborated by *in vitro* models: studies using human PDLSCs under LPS or TNF-α-induced inflammatory stress, as well as macrophages infected with *P. gingivalis*, have similarly shown reduced PINK1/Parkin levels and impaired mitophagy (61, 62).

The impact of PINK1-mediated mitophagy on periodontitis progression appears to operate through mitochondrial-dependent apoptotic pathways (63) or inflammatory regulation (64). In apoptosis regulation, PINK1 has been demonstrated to modulate the expression of apoptosis-related genes including Bax, Bcl-2, and caspase-9 (65). Enhanced PINK1 expression effectively suppresses stimulus-induced apoptosis in periodontal cells (66, 67). Regarding inflammatory control, in oral epithelial cells exposed to *P. gingivalis*, increased mtROS activates AMP-activated protein kinase, resulting in tuberous sclerosis complex (TSC) 2 phosphorylation and TSC1/TSC2 complex formation, inhibiting mechanistic target of rapamycin complex 1, activating ULK1, and inducing mitophagy (68). This phenomenon is also observed in ischemia-reperfusion injury (IRI) (69), lending support to the existence of common mechanisms of impaired MQC across different diseases. The loss of mitophagy exacerbates the release of mtROS and oxidative stress, thereby compromising the health of periodontal cells (70). Furthermore, PINK1/Parkin pathway plays a crucial role in balancing bone remodeling in periodontitis (71, 72). PINK1/Parkin-mediated mitophagy enhances the osteogenic differentiation potential of PDLSCs-derived single-cell colonies (67). The clearance of oxidative damage by mitophagy has also been confirmed to promote the osteogenic differentiation of BMSCs (73).

Modulating mitophagy to decrease oxidative stress or enhance osteogenic differentiation may thus be a viable therapeutic approach for periodontitis. Although experimental models have confirmed that inflammation can lead to impaired mitophagy, this finding requires further thorough evaluation at multiple levels to demonstrate that it is an independent event.

### 3.4 Effects of mitobiogenesis on periodontitis

mtDNA is the only genetic material outside the nucleus and is highly susceptible to mutations due to the ROS-rich mitochondrial environment and the lack of efficient DNA repair mechanisms. In periodontitis, mtDNA abnormalities can be categorized into four distinct layers.

The first involves changes in mtDNA copy number. Human studies have demonstrated the presence of reduced mtDNA content and large-scale deletions in the gingival tissues of periodontitis patients (74–77). These findings revealed a relationship between aggressive periodontitis and mitochondrial gene polymorphisms, providing a genetic perspective for understanding the etiology of periodontitis.

Another aspect concerns structural mutations and deletions. mtDNA deletions have been detected in gingival tissues from periodontitis patients (78, 79). Unlike copy number changes, these structural alterations represent permanent genetic changes that may predispose individuals to heightened disease susceptibility. Moreover, mtDNA deletions and mutations contribute to mitochondrial dysfunction, which has been identified as a key factor exacerbating periodontal tissue damage in the context of diabetes (80).

In parallel, oxidative damage to mtDNA represents a third layer of abnormality. Due to its proximity to the electron transport chain and limited protective histones, mtDNA is particularly vulnerable to oxidative attack. Oxidized mtDNA bases impair replication and transcription, further compromising mitochondrial function in a self-amplifying cycle (81).

Beyond these alterations within mitochondria, the release of mtDNA into the cytosol and extracellular space activates innate immune responses. As a damage-associated molecular pattern (DAMP), mtDNA—which contains CpG motifs similar to those found in bacterial DNA—is recognized by pattern recognition receptors such as cGAS-STING and TLR9, thereby triggering innate immune responses (82, 83). After TLR9 recognizes CpG DNA, it triggers an inflammatory response by activating the MAPK and NF-κB pathways (34). Current understanding suggests that ROS can trigger the opening of the mitochondrial permeability transition pore (mPTP) by inducing the oligomerization of voltage-dependent anion channel 1 and the mitochondrial ATP-sensitive potassium channel (84, 85). Upon leaking into the cytoplasm via the mPTP (51), mtDNA initiates and exacerbates inflammation (86). The accumulation of mtROS and mtDNA intensifies periodontal inflammation, activates the nucleotide-binding oligomerization domain-like receptor protein 3 (NLRP3) inflammasome, and accelerates tissue breakdown (87).

Beyond these mtDNA abnormalities, mitochondrial biosynthesis is jointly regulated by the nuclear genome, among which the key molecules include: PGC-1α nuclear respiratory factor-1/2 (Nrf1/2) and mitochondrial transcription factor A (TFAM) (88). In the periodontal tissues of the periodontitis animal model, the expression levels of factors such as PGC-1α were detected to be decreased, which aggravated the functional disorder of cells (89). Similar findings were found in elderly patients with periodontitis (90). The application of strong antioxidants in rats with diabetic periodontitis reduces periodontal damage, increases the expression of PGC-1α, and enhances mitochondrial biosynthesis (91).

Mitochondrial dysfunction (including impaired biogenesis, mtDNA instability, and defective quality control) plays a central role in periodontitis pathogenesis, yet key mechanisms remain elusive and require validation in more physiologically relevant models.

## 4 Linking mitochondrial dysfunction to cardiovascular pathogenesis

**Figure 5:**
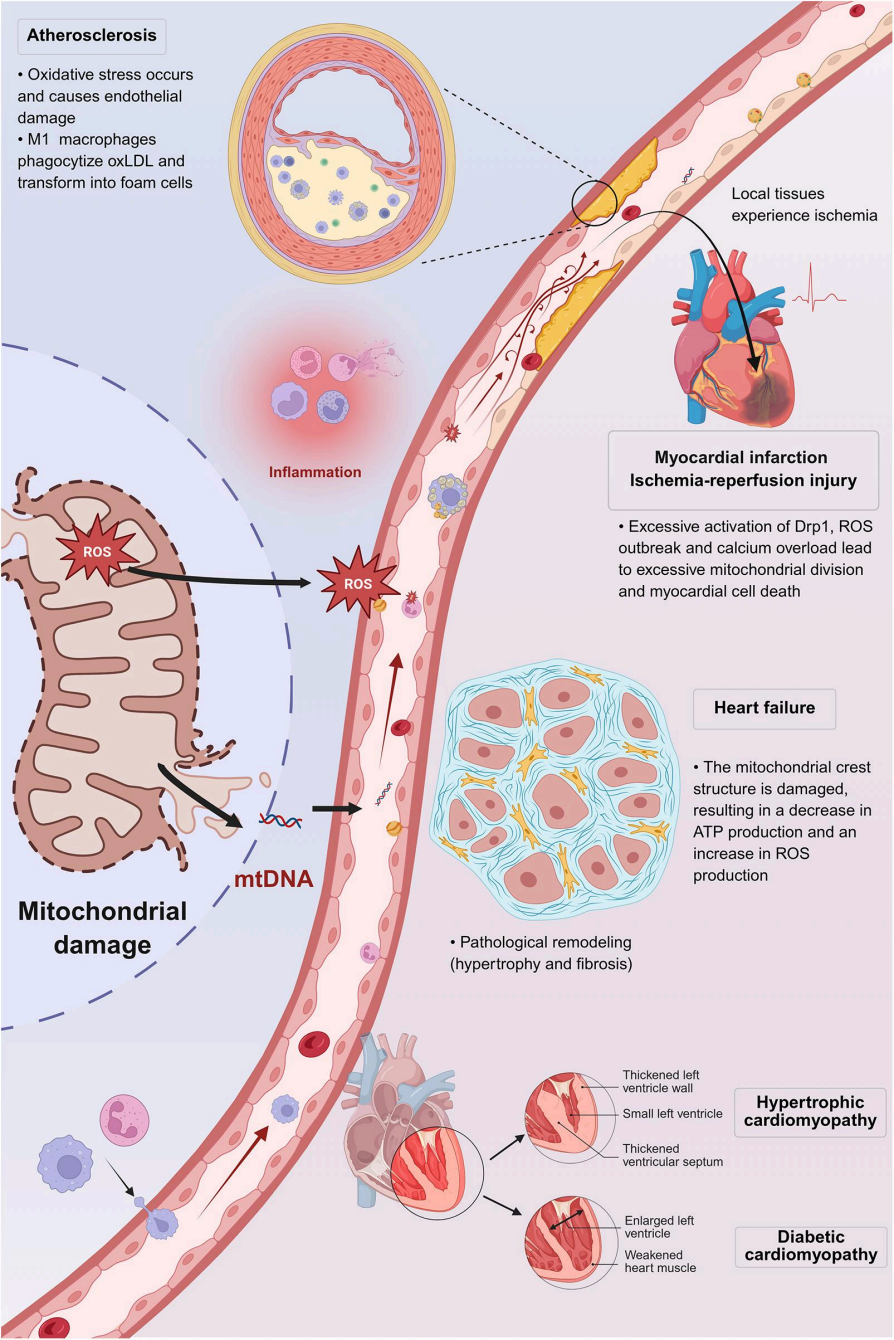
The key role of mitochondrial dysfunction in cardiovascular pathogenesis. Mitochondrial damage is a key mechanism in cardiovascular diseases. It often begins with endothelial dysfunction triggered by risk factors such as oxidative stress, which leads to increased ROS production and inflammation. Key disease processes include: (1) Atherosclerosis: macrophages take up oxidized LDL, forming foam cells (52). (2) Myocardial infarction/ischemia-reperfusion: excessive Drp1 activity, ROS bursts, and calcium overload causes pathological fission and cardiomyocyte death (92). (3) Heart failure: disrupted cristae structure impairs energy production, increases oxidative stress, and promotes tissue remodeling (hypertrophy/fibrosis) (93). This mechanism also underlies hypertrophic and diabetic cardiomyopathy. (Created in BioRender).

While periodontitis and CVD affect distinct organ systems, growing evidence suggests that mitochondrial dysfunction may serve as a potential molecular bridge linking these two chronic conditions. As a pathological hallmark of periodontitis, mitochondrial dysfunction and its mechanisms, including oxidative stress, impaired autophagy, and disrupted energy metabolism, have been discussed in previous sections. A subsequent question is whether these organelle pathologies also contribute to the pathophysiology of the remote cardiovascular system (Figure 5).

### 4.1 ROS-induced ROS release in cardiovascular dysfunction

In cardiovascular biology, oxidative stress originates from multiple sources that operate in hierarchical cascade. The cascade begins with NADPH oxidases, particularly NOX2 and NOX4, which serve as primary ROS generators in the cardiovascular system (94). These enzymes respond rapidly to pathological stimuli such as angiotensin II, shear stress, and inflammatory cytokines, producing superoxide and hydrogen peroxide as initial signaling molecules (95). This initial ROS burst is tightly regulated and represents the first wave of oxidative stress in response to injury.

This initial wave triggers a secondary more sustained wave of ROS generation from mitochondria through a phenomenon termed “ROS-induced ROS release” (96).Within mitochondria, complex I has been identified as a primary source of superoxide bursts (97). Under pathological conditions, damaged mitochondria initiate this cascade, leading to loss of mitochondrial membrane potential and exacerbation of endothelial dysfunction. This self-amplifying cycle further exacerbates oxidative stress and increases the risk of subsequent cardiovascular events. Excessive mtROS not only oxidize low-density lipoprotein (LDL) to promote foam cell formation but also drive signaling pathways mediated by NF-κB, hypoxia-inducible factor-1α, and the NLRP3 inflammasome to foster sustained endothelial inflammation (52, 98). Inhibiting the activity of complex I during heart reperfusion after infarction may protect cardiomyocytes against mtROS-mediated injury to some extent (98, 99). These current evidence remains experimental, and translating these strategies into clinical practice still faces significant challenges.

Simultaneously, ROS-induced damage to mitochondrial components establishes a self-amplifying pathological circuit. The oxidative damage inflicted during these stages attacks mtDNA, which lacks protective histones and has limited repair capacity. A new self-amplifying pathological circuit is thus formed: mutations and functional decline of the mitochondrial genome feed back to enhance ROS generation (81). When oxidative stress overwhelms MQC mechanisms, mtROS, oxidized mtDNA fragments and apoptosis signal released into the cytosol via the mPTP and triggered subsequent pathological processes (100, 101).For example, reduced cardiolipin levels and increased lipid peroxidation levels have been reported in murine models of I/R injury (102, 103). Oxidized cardiolipin is a proinflammatory signal that stimulates the secretion of cytokines and adhesion molecules (102). Additionally, ROS-induced mtDNA damage participates in atherogenesis. This has been confirmed in both ApoE^−^/^−^ mice and human atherosclerotic specimens, and associated with plaque instability (104).These findings highlight the importance of crista integrity and ROS regulation in CVD, indicating potential therapeutic targets. The current findings are predominantly derived from oversimplified disease models that fail to recapitulate the chronic and multifactorial nature of human CVD

Once released, mtDNA acts as a DAMP that activates downstream pattern recognition receptors, particularly the NLRP3 inflammasome and the cGAS-STING pathway (101). This activation triggers the maturation of IL-1β and IL-18, amplifying local and systemic inflammation (105). In the cardiovascular context, this cascade promotes endothelial activation, monocyte recruitment, foam cell formation, and atherosclerotic progression (106).

Like periodontal cells, changes in the number and integrity of mitochondrial cristae affect the function of cardiovascular system. Evidence from animal models demonstrates that cardiomyocytes from heart failure (HF) models in mice and dogs, as well as ECs from rat pulmonary hypertension models, exhibit impaired cristae structure, vacuolization, and other structural abnormalities (107–109). In human studies, vacuolated mitochondria have been reported in cardiomyocytes from patients with ischemic heart disease (110), and alterations in cristae have been detected in ECs from human atherosclerotic plaques (111). By decreasing respiratory chain efficiency and increasing ROS generation, these structural defects provide functional support for the causal role of cristae abnormalities (Figure 4).

### 4.2 Mitochondrial dynamics in the pathogenesis of CVD

Imbalanced mitochondrial fission and fusion contribute significantly to CVD progression. The evidence from murine models demonstrates that the pathological stimuli, such as ischemia or high-fat diet, promote Drp1-dependent fission, which is associated with increased ROS production, Ca^2+^ overload, and ultimately cardiomyocyte death (112, 113). The occurrence of IRI during the treatment of acute myocardial ischemia (AMI) remains an formidable clinical challenge. At present, studies in rat models have demonstrated that ceria nanoparticles can alleviate AMI damage by suppressing the generation of phosphorylated Drp1 (114). Similarly, the impairment of fusion is equally detrimental. According to previous animal intervention studies, suppressing OPA1 expression results in increased mitochondrial fragmentation, abnormal mitochondrial function, and cardiomyocyte apoptosis (115–117). In contrast, overexpression of OPA1 improves mitochondrial function and reduces cardiac damage after IRI (118). Further evidence in humans, mutations in the OPA1 gene have been linked to atherosclerosis, dilated cardiomyopathy, and cardiac hypertrophy (117, 119, 120). Other regulators, including Oma1 and Omentin1, also modulate OPA1, Mfn2, and Drp1 activity (121, 122).

Importantly, the disruption of mitochondrial dynamics forms a vicious cycle with other pathological factors. Excess ROS and Ca^2+^ not only result from but also exacerbate dynamical imbalance. Experimental murine models show that Mfn2 inhibition increases mitochondrial Ca^2+^ uptake, while triggers mPTP opening, thereby exacerbating cardiomyocyte apoptosis in IRI (123). The interplay underscores that mitochondrial dynamics are not an isolated function but are integrated into the broader pathological network of CVD.

### 4.3 Mitophagy dysregulation in CVD

Impaired mitophagy compromises MQC and promotes CVD progression (124). This pathological phenotype is mechanistically linked to the impaired clearance of damaged cellular components. Previous studies have demonstrated that the mitophagyis suppressed in various cardiovascular conditions, including diabetic cardiomyopathy, MI, and HF (125). In model mice at different growth stages, the deletion of either PINK1 or Parkin can lead to the emergence of cardiac pathologies (126–128). These studies suggest that mitophagy exhibits significant developmental stage and age specificity. Furthermore, in *Drosophila melanogaster*, the deletion of Parkin causes a dilated cardiomyopathy, which was rescued by cardiomyocyte-specific re-expression of Parkin (129). BNIP3L/NIP3-like protein X (NIX) is another pathway that can recruit PINK1 to mitochondria and induce mitophagy. Knockout of either the BNIP3L or BNIP3 gene leads to spontaneous cardiac enlargement and contractile dysfunction in mice (130). In addition, in diabetic mouse models, BNIP3 was inhibited, thereby losing its function of protecting blood vessels under IRI (131). The detrimental consequences of mitophagic failure can extend to inflammatory activation. Recent research shows that mitophagy deficiency causes mtDNA accumulation, interfering with minichromosome maintenance complex component 8 separation, overactivating the cGAS–STING pathway, and increasing the risk of coronary vasculitis (132). These findings underscore that precise spatiotemporal regulation of mitophagy is paramount for cardiovascular health—a level of complexity that current experimental models often struggle to capture.

### 4.4 Dysfunctional mitobiogenesis in cardiovascular pathology

mtDNA damage is not only a biomarker of CVD but also a significant driver of its pathology (133). In existing cardiovascular research, mtDNA abnormalities can be understood through several mechanistically distinct layers.

The first concerns mtDNA copy number alterations. Reduced mtDNA copy number (mtDNA-CN) is significantly associated with atherosclerosis, cardiovascular events, and all-cause mortality (134, 135). Multiple cohort studies have demonstrated that mtDNA-CN correlates with age, frailty, and increased mortality risk, serving as an independent predictive biomarker (136). Furthermore, changes in mtDNA-CN interact with nuclear DNA methylation patterns, suggesting an epigenetic regulatory network linking mitochondrial and nuclear genomes in cardiovascular disease development (137).

Another layer involves mtDNA deletions and structural mutations. In atherosclerotic lesions, mtDNA mutation burden progressively increases from normal intima to fibrous plaques and lipid-rich plaques, indicating a close association with disease progression (138, 139). The heterogeneous distribution of mtDNA mutations may partly explain the focal and non-uniform characteristics of atherosclerotic lesions (140). Notably, mtDNA mutations at different loci may exert opposing effects on atherosclerosis depending on their impact on mitochondrial ROS generation capacity (141).

Oxidative mtDNA damage has been demonstrated to contribute to atherosclerotic progression. Studies have shown that aortic tissue, hearts, and circulating leukocytes from atherosclerosis patients is associated with significantly more mtDNA damage compared to healthy controls (142, 143). Consistent with the above, such damage predominantly caused by oxidative stress rather than genetic factors. oxLDL-induced mtDNA damage in vascular smooth muscle cells triggers a cascade of events: loss of mitochondrial membrane potential, upregulation of PINK1, and inhibition of complex I production (104). These changes enhance mitophagy, impair mitochondrial respiration, and ultimately compromise the fibrous cap structure that stabilizes plaques (104). Mitochondria-targeted antioxidants can effectively reduce mtDNA oxidative damage, decrease intraplaque macrophage proliferation, and slow atherosclerotic progression (144).

Once released, mtDNA serves as a pro-inflammatory signal, appearing in the process of cardiovascular tissue remodeling. The cGAS acts as the principal signal transducer for mtDNA, initiating a downstream signaling cascade (145). This pathogenic role is demonstrated by interventional studies in murine MI studies, wherein inhibition of either cGAS itself or its key effector molecules, attenuates pathological remodeling and preserves cardiac function (146, 147). In addition, the released mtDNA can also induce pyroptosis in cells through the NLRP3 inflammasome, amplifying the local inflammatory response (148); or activate the TLR9 pathway to mediate the inflammatory cascade mediated by NF-κB, further promoting endothelial dysfunction and plaque instability (149).

Beyond mtDNA homeostasis, crosstalk between mitochondria and the nucleus regulates the cellular environment. Crosstalk between mitochondria and the nucleus regulates the cell environment. As a key regulator of mitobiogenesis, PGC-1*α* coordinates the binding of TFAM to mtDNA by initiating the expression of Nrf1 and Nrf2, thereby facilitating mitobiogenesis (150, 151). PGC-1α enhances energy synthesis by upregulating the expression of respiratory chain components and increasing the activity of ATP/ADP transporters (152). Among these mechanisms, Nrf is associated with the expression of nuclear genes encoding subunits of the respiratory complexes (153). In response to injury, Nrf1 triggers transcriptional reprogramming in cardiomyocytes, promoting regeneration in neonatal mice and mitigating IRI in adult mice (154). Moreover, Nrf1 serves as a transcription factor in antioxidant defense mechanisms, mitigating oxidative stress and maintaining cardiovascular homeostasis (155). Through these mechanisms, PGC-1α plays a crucial role in mitobiogenesis, oxidative metabolism, and the control of inflammation, underscoring its potential as a therapeutic target in CVD.

## 5 Inter-organelle communication and systemic crosstalk

In preceding sections, we have elucidated how mitochondria and the nucleus communicate through key regulators such as PGC-1α, Nrf1/2, coordinately directing critical cellular processes. In addition to direct nuclear communication, mitochondria also interconnect with other organelles to translate nuclear instructions into specific cellular behaviors. Strikingly, approximately 20% of the mitochondrial surface is in close apposition to the ER, suggesting a functional coupling between them (156). This mutual structure, termed Mitochondria-Associated ER Membranes (MAMs), mediates the exchange of lipids and Ca^2+^ (157). In cardiomyocytes, precise Ca^2+^ transfer via MAMs is crucial for matching energy production with contraction requirements (158, 159). Furthermore, Mfn2 serves as a key molecular component in maintaining MAMs structural integrity (160). Consequently, disruption of MAMs architecture—including deficiency in Mfn2—along with the subsequent Ca^2+^ overload and mPTP opening, may collectively contribute to impaired cardiac function (50). Mitochondrial fission requires coordinated involvement of multiple organelles, including the ER, Golgi apparatus, and lysosomes. In the initial phase of fission, the ER wraps around mitochondria to establish the primary constriction site and recruits Drp1 for oligomerization (161, 162). Subsequently, vesicles derived from the Golgi apparatus or lysosomes transport phosphatidylinositol 4-phosphate to this site, where it activates the actin cytoskeleton system to further deepen membrane constriction (163, 164). Ultimately, the FIS1–TBC1D15–Rab7 pathway facilitates the clearance of vesicular obstacles, enabling Drp1 to execute membrane scission (161, 165, 166). The complete interactome underlying this highly regulated process remains to be fully elucidated.

The metabolic and signaling microenvironment established through inter-organellar interactions directly shapes the functional states of immune cells. As tissue-resident immune cells, macrophages can undergo phenotypic polarization in response to various stimuli (167, 168). Among these, M1 polarization is accompanied by significant mitochondrial metabolic reprogramming, shifting cellular energy production from OXPHOS to glycolysis (169–171). This metabolic switch not only facilitates rapid energy supply but also enhances bactericidal functions through the generation of metabolic byproducts and an acidic microenvironment (172). mtROS and mtDNA play key roles in macrophage immune responses: mtROS drives M1-associated inflammatory reactions (173), while mtDNA can activate the NLRP3 inflammasome (174), thereby exacerbating inflammatory cascades. Under pathological conditions, mitochondrial dysfunction promotes M1 macrophage polarization, which in turn aggravates inflammation and bone resorption in periodontitis (35, 175). A similar mechanism operates in atherosclerosis, where elevated glycolytic activity accelerates lipoprotein oxidation and aggregation, while also enhancing lipoprotein uptake by M1 macrophages, thereby driving disease progression (176). Neutrophils participate in innate immune defense through phagocytosis, degranulation, and the formation of neutrophil extracellular traps (NETs) (177). Among these mechanisms, mtDNA plays a role in the formation of NETs by binding to antimicrobial proteins (36). It is released via vesicles to form a fibrous reticular structure that captures pathogens (36). Certain bacterial virulence factors, such as *P. gingivalis* peptidylarginine deiminase, can assist pathogens in evading NET-mediated killing (178). A key feature of NETs is their ability to capture periodontopathogens without necessarily inhibiting bacterial viability (179). The protective efficacy of NETs remains controversial, and their exposure of self-DNA may exacerbate tissue damage and immune dysregulation.

Beyond direct cellular mechanisms, an emerging concept—mitochondria-gut microbiota crosstalk—is gaining increasing research attention (180). Evidence indicates that periodontitis can alter the gut microbiota through oral pathogens and inflammatory mediators (181). This gut ecological disruption exacerbates systemic inflammation in turn. Specific gut microbial metabolites, such as short-chain fatty acids, have been shown to modulate mitochondrial function in distant host organs (182, 183).

## 6 Mitochondria-derived mediators in oral-cardiovascular crosstalk

In this review, the mechanistic role of mitochondrial dysfunction between the two diseases is primarily established on association rather than causality, relying on observed similarities in mitochondrial phenotypic alterations. Direct evidence remains lacking to demonstrate that periodontitis-originated damage triggers mitochondrial dysfunction that in turn initiates remote cardiovascular pathology, or vice versa. Furthermore, shared risk factors like aging, obesity, and smoking—which are potent mitochondrial stressors—may drive the dysfunction observed in both diseases, as these risk factors are themselves potent inducers of mitochondrial damage. Rather than acting in isolation, periodontitis or CVD may exert synergistic effects with such pre-existing mitochondrial vulnerability induced by shared risk factors. Current study designs often fail to adequately dissociate these confounding variables during model establishment, potentially leading to overinterpretation of the disease effects attributable solely to mitochondrial dysfunction.

In parallel, a discussion of potential mediating factors may facilitate a deeper understanding of direct causal inter-organ signaling mechanisms. As outlined in Section 3.4, mtDNA containing CpG motifs is released during periodontitis. Once in the circulation, it can act as a DAMP that activates innate immune pathways, potentially contributing to systemic inflammation and promoting cardiovascular events. Although clinical correlations between circulating mtDNA levels and periodontal disease severity have been reported, direct evidence that periodontitis-induced mtDNA release actively drives cardiovascular pathology remains to be validated in experimental models.

Extracellular vesicles (EVs) have recently emerged as important mediators of intercellular communication (184, 185). Three types of mitochondrial components have been identified within EVs: free mtDNA, functional mitochondria, and mitochondrial contents (186). In the context of periodontitis, macrophage-derived EVs enriched with mitochondrial components have been shown to exacerbate periodontal bone loss (187). It indicates that EVs are indeed present and biologically active in periodontal inflammation. In parallel, bacterial outer membrane vesicles serve as carriers for fimbriae, endotoxins, and proteases, contributing to endothelial dysfunction and vascular injury following infection by periodontal pathogens (188, 189). No direct evidence yet shows that periodontitis-derived, mitochondria-containing EVs reach the cardiovascular system. Nevertheless the available evidence strongly suggests that EVs-mediated mitochondrial signaling may serve as a potential pathway between periodontitis and CVD.

Beyond the mechanisms discussed above, mitochondrial stress signals originating from periodontal tissues can induce immunometabolic reprogramming. Previous sections of this review have summarized evidence for metabolic reprogramming, specifically the shift from oxidative phosphorylation to glycolysis and polarization of macrophages. These changes constitute a local foundation for immune cell functional remodeling. Building on this, recent studies suggest that chronic inflammation triggered by periodontitis may further act on the bone marrow, inducing functional reprogramming of hematopoietic stem and progenitor cells (190, 191). These cells undergo changes in metabolism and epigenetics, producing more pro-inflammatory monocytes and neutrophils (191). This may serve as a mechanism linking periodontitis to CVD. Clinical imaging studies provide supporting evidence: metabolic activity in periodontal tissues correlates positively with bone marrow hematopoietic activity and arterial inflammation (192). This suggests that immune reprogramming in the bone marrow may serve as an important link between periodontitis and CVD. Collectively, however, direct evidence supporting the involvement of immune reprogramming in linking periodontitis to CVD through mitochondrial dysfunction remains limited and requires further investigation.

## 7 Future perspectives

The majority of evidence originates from cell cultures or animal models induced by single pathogens, which largely overlook the complex metabolic networks in living organisms, as well as the heterogeneity and compensatory capacity of mitochondrial function. *P*. gingivalis is widely employed as a model pathogen for constructing periodontitis models due to its well-characterized virulence factors, ease of manipulation *in vitro*, and consistent induction of characteristic periodontitis pathological features (193). Periodontitis is fundamentally a disease driven by polymicrobial synergism (194). While the current research centered on a single pathogen holds significant value, it may be insufficient to fully elucidate the comprehensive landscape of host-microbiota interactions, thereby presenting potential limitations in understanding the disease mechanisms. Additionally, mitochondrial functions exhibit dual roles, and their context-dependent manifestations in preclinical models hinder an objective assessment of the underlying mechanisms. The precise pathways through which localized mitochondrial damage is converted into systemic signals and crossed physiological barriers to remote tissue injury remain unknown.

The therapeutic potential of current mitochondrial-targeting strategies appears overestimated in existing research. Various strategies for mitochondrial dysfunction have been developed, including antioxidants (195, 196), regulation of mitochondrial dynamics (197), mitochondrial, autophagye, calcium homeostasis (198), and mitochondrial transplantation (199–202), the related treatments still face challenges such as uncertain efficacy, side effects and application limitations.

To address these challenges, we propose several strategic directions for future research. First, there is a need to develop animal models that concurrently simulate periodontitis and CVD, coupled with targeted tools to reverse mitochondrial dysfunction at specific disease stages to determine if this halts disease progression. Second, we should focus on identifying mitochondria-specific, quantifiable biomarkers detectable in blood. Large-scale clinical cohorts are needed to prospectively validate the prognostic and predictive value of those biomarkers for diseases risk. Third, research should explore the carriers responsible for mitochondrial signaling along the “oral-cardiovascular” axis and investigate how their behavior is modulated by disease states. Fourth, innovative drug delivery systems that enable controlled release of mitochondrial-targeted agents in response to specific pathological microenvironments—such as locally elevated ROS.

## 8 Conclusion

Mitochondria play a crucial role in maintaining health in both the periodontal and cardiovascular systems; however, their dysfunction significantly contributes to disease development. This dual nature makes them important targets for therapeutic interventions. Although oxidative stress, defective quality control, and metabolic dysregulation are well-established hallmarks of periodontitis and CVD, the specific pathways linking these two disease entities through common mitochondrial mechanisms are still poorly understood. Significant knowledge gaps still persist, including monitoring real-time changes in the redox balance, improving the precision of antioxidant therapies, and establishing long-term outcome data and standardized effectiveness criteria for mitochondria-targeted interventions. Future studies should prioritize elucidating how mitochondrial structure, function, and signaling change over time when both disease entities are present. Interdisciplinary collaboration will be essential to address these challenges, enabling the development of accurate diagnostic tools and precise therapeutic interventions that target mitochondrial dysfunction as a central driver of the oral–systemic disease relationships.
